# Cultural adaptation of the facial emotion perception test for use in Zimbabwe: A pilot study

**DOI:** 10.4102/sajpsychiatry.v31i0.2434

**Published:** 2025-05-28

**Authors:** Denford Gudyanga, Primrose Nyamayaro, Summer Frandsen, Rebecca Easter, Sarah Derveeuw, Pauline Thibaut, Alina Dillahunt, Conall O’Cleirigh, Leah Rubin, Scott A. Langenecker, Melanie Abas

**Affiliations:** 1 Department of Mental Health, Faculty of Medicine and Health Sciences, University of Zimbabwe, Harare, Zimbabwe; 2 Ohio State University, Columbus, United States; 3 Department of Psychology, University of Illinois, Chicago, United States; 4 Department of Health Services and Population Research, Institute of Psychiatry, Psychology and Neuroscience, King’s College London, London, United Kingdom; 5 Massachusetts General Hospital, Harvard Medical School, Boston, United States; 6 Department of Neurology, John Hopkins University, Baltimore, United States; 7 Wexner Medical Center, Faculty of Psychiatry and Behavioral Health, Ohio State University, Columbus, United States

**Keywords:** depression, adaptation, psychological test, Facial Emotion Perception Test, negative valence, research domain criteria, Zimbabwe

## Abstract

**Background:**

In African countries, including Zimbabwe, about half of those with depression respond to first-line therapies like problem-solving therapy. Predicting who needs more intensive treatment is challenging. In the US and Europe, tools like the Facial Emotion Perception Test (FEPT) help match treatments to likely responders. However, its applicability in Zimbabwe is unexplored.

**Aim:**

To develop a racially diverse adaptation of the FEPT for Shona-speaking Zimbabweans.

**Setting:**

Outpatient primary healthcare clinics at Marondera Provincial Hospital and Chitungwiza Central Hospital, Zimbabwe.

**Methods:**

Facial Emotion Perception Test was adapted using the Ecological Validity Model’s eight constructs through a four-step process: expert consultation, preliminary content adaptation, iterative content adaptation, and finalising adaptation. Three focus groups and 12 cognitive interviews assessed cultural appropriateness, suitability, usability and acceptability of FEPT for Zimbabwean Shona speakers. Fifteen participants, including graduates, primary healthcare workers and individuals with lived experience of depression, took part.

**Results:**

Key adaptations of FEPT-Multiple-Races (MR)-Shona include: (1) added 20 black and 20 Asian face stimuli for cultural relevance; (2) improved stimuli resolution for clarity; (3) extended test duration from 6 to 10 minutes for repeatable tutorials; (4) provided bilingual instructions in Shona and English; (5) shifted to a low-cost touchscreen tablet, familiar to Zimbabwean participants.

**Conclusion:**

The adaptation shows promising cultural relevance and usability for Shona speakers. Further testing with diverse educational and contextual backgrounds is needed to enhance cross-cultural and ecological validity.

**Contribution:**

This study highlights the importance of culturally adapting cognitive performance tools that can potentially improve depression treatment outcomes in low-income countries.

## Introduction

Depression affects an estimated 4.5% of people in African countries and is a major contributor to disability and illness.^1^ In Zimbabwe, depression is estimated to cause productivity losses of $154.8 million, equivalent to 0.6% of Zimbabwe’s gross domestic product (GDP), because of absenteeism, presenteeism, premature death and disability.^2^

In African countries, including Zimbabwe, about half of people with depression respond to simple first-line therapies such as problem-solving therapy and peer group support.^3,4^ Given that a significant proportion of individuals do not respond to these low-intensity interventions, identifying which patients are most likely to benefit from more complex psychological therapies or antidepressant medication should be a priority. Tailoring treatment in this way would ensure the most economical and resource-efficient use of limited healthcare resources, maximising the impact of mental health interventions in settings with constrained resources.

In low-resource settings including Zimbabwe, response to depression treatment is assessed by self-reported symptoms, using tools such as the Patient Health Questionnaire (PHQ-9).^5,6^ In contrast, in some settings in the United States (US) and Europe, changes in emotion processing have been shown to predict and match depression treatments to those most likely to respond.^7,8^ Changes in emotion processing in depression are reflected in disruptions in brain systems of negative valence including the amygdala, subgenual cingulate and insula, and can predict depression treatment response.^9,10,11,12,13^ A test commonly used is the Facial Emotion Perception Test (FEPT), a short, computerised visual test that assesses the speed and accuracy of categorising facial emotion expressions.^10,14^

Facial emotion perception deficits are well-documented in individuals with depression and have been associated with symptom severity, social dysfunction and cognitive-affective processing impairments.^15,16,17^ Emerging evidence suggests that facial emotion perception tests, including the FEPT, may have predictive utility in assessing treatment response and clinical outcomes in depression. Studies have found that individuals with greater deficits in emotion recognition, particularly in identifying positive emotions such as happiness, may exhibit poorer responses to antidepressant treatment.^18,19^ Additionally, improvements in facial emotion recognition have been observed in patients undergoing psychotherapy and pharmacological interventions, suggesting that changes in this cognitive-affective domain may serve as a marker of treatment efficacy.^20,21^

Despite these promising findings, research on the predictive value of the FEPT in depression treatment response remains limited, particularly in non-Western populations. While numerous versions of the FEPT have been developed in high-income countries,^22^ there is a gap in knowledge about their feasibility in low- and middle-income countries (LMICs) like Zimbabwe. A recent systematic review found limited research on cognitive performance tests as predictors of depression treatment response in LMICs, other than the use of memory tests.^23^ In addition, the relevance of actual stimuli used (matching race and sex) can have profound effects on how the skill is measured (see race bias). There is therefore a need to culturally adapt tests like the FEPT, originally designed in the US and Europe, for use in Zimbabwe. Such tools could facilitate research on stratified care for depression in African countries like Zimbabwe.

Given the need to culturally adapt tests like the FEPT for use in Zimbabwe, the Ecological Validity Model (EVM) by Bernal et al.^24^ offers a robust framework to guide this process. The EVM emphasises eight key dimensions: language, persons, metaphor, content, concepts, goals, methods and context. Language adaptation ensures that the test is linguistically appropriate for the target population.^24,25^ The ‘persons’ dimension considers the cultural characteristics of the individuals involved. Metaphor adaptation involves aligning culturally relevant symbols and analogies. Content adaptation ensures that the material is culturally relevant and meaningful. Concepts adaptation addresses the cultural understanding of the ideas being tested. Goals adaptation aligns the test’s objectives with the cultural values and priorities of the target population. Methods adaptation involves modifying the procedures to fit the cultural context. Finally, ‘context’ adaptation ensures that the test setting reflects the everyday environment of the target population.^24^ By addressing these dimensions, the EVM improves test reliability by ensuring that the test measures what it is intended to measure across different cultural contexts. This reduces biases and errors that may arise from cultural misunderstandings or misinterpretations, thereby enhancing the accuracy and consistency of the test results. The model also tackles several cultural adaptation challenges. These include overcoming language barriers, ensuring cultural relevance of test content and addressing different cultural interpretations of concepts and metaphors. Additionally, it helps align the test goals with the cultural values of the target population and adapts the methods to fit the cultural context. By resolving these challenges, the EVM ensures that the adapted tests are both culturally sensitive and valid, enhancing their applicability and accuracy across different cultural settings.

## Aim

The aim was to develop a racially diverse adaptation of the FEPT for use with Shona-speaking people, who constitute approximately 80% of Zimbabwe’s population.^26^

## Methods

### Setting

The adaptation work was conducted in outpatient primary healthcare clinics at Marondera Provincial Hospital and Chitungwiza Central Hospital. Marondera, the capital of Mashonaland East province, has a semi-rural population of about 224 000, and Chitungwiza is an urban town with five townships and around 391 000 residents.^27^

### Participants

Participants were purposively recruited to include a mix of educational, gender and health characteristics. Participants comprised graduates with a background in psychology, counselling, and social work, primary health workers and people with lived experience of depression, many of whom were also living with human immunodeficiency virus (HIV). Their insight into future Zimbabwean FEPT users’ cultural and educational background was needed to inform the cultural adaptation with a focus on suitability and usability of the test. Including people living with HIV was important in the context of research in Zimbabwe, given the burden of HIV, and the high prevalence of depression in adults living with HIV. The known interactions between HIV, depression and cognitive-affective processes,^28^ including emotion perception^28,29,30^ highlight the importance of including this subgroup to ensure the test’s applicability across diverse health backgrounds.

The participant distribution was purposively designed to focus on lived experiences of depression and contact with or experience working with people who use primary health care services, including treatment for depression and HIV in both urban and rural areas. Special attention was given to participants’ familiarity with computer-based cognitive tests and general computer literacy. All graduates, one primary health care worker and two individuals with lived experience reported average to high computer literacy and low to average familiarity with cognitive or computer-based cognitive tests. Three primary health care workers had low computer literacy levels and no familiarity with computer-based cognitive tests. Five individuals with lived experience had low to no computer literacy and no familiarity with cognitive or computer-based cognitive tests.

This approach ensured diverse perspectives in the adaptation process. Graduates in psychology, counselling and social work provided theoretical and professional insights into emotion perception, while primary health workers contributed practical knowledge relevant to frontline healthcare. Including individuals with lived experience of depression was crucial for ensuring that the adapted test accurately reflected the target population’s emotional expressions and interpretations. This purposive sampling balanced professional expertise with real-world applicability, enhancing the cultural validity of the adapted test. Although not statistically representative, this approach aimed to improve the cultural and ecological relevance of the adapted test. Future studies are needed to evaluate the psychometric properties of the adapted version in a larger, more representative sample.

### Original facial emotion perception test

The original FEPT, developed by Langenecker et al. in 2002 and 2005, is a 6-min computerised assessment designed to measure the speed and accuracy of categorising facial emotional expressions.^14^ This test uses black-and-white photographs of white faces from the Ekman set,^31^ and it includes five animal stimuli (dog, cat, bird, fish, monkey and/or primate) as control images.^14^ Participants assign the presented facial expressions to one of five emotion categories: angry, happy, fearful, sad or neutral through a computer keyboard. Each facial expression stimulus is displayed for 300 ms, followed by a 100-ms grey-scale mask.^14^ Participants then have 2600 ms to choose the correct emotion from the five options provided.^14^

### Adaptation research team

The research team consisted of three Zimbabwean and three international mental health researchers with expertise in psychiatry, neuropsychology, adapting mental health measurement tools into the Zimbabwean Shona language, and use of the FEPT in multiracial groups in the US and Eastern Europe.

### Adaptation methods

The adaptation utilised the eight constructs of the Ecological Validity Model: language, persons, metaphor, content, concepts, goals, methods and context.^24^ The adaptation followed a modified version of Sit et al.’s,^32^ four-step process that includes: (1) stage setting and expert consultation, (2) preliminary content adaptation, (3) iterative content adaptation with community members and (4) finalising adaptation.^32^

Three focus group discussions (FGDs),^25^ and 12 cognitive interviews,^33^ with participants and the research team were conducted to inform the FEPT adaptation. These qualitative data-collection methods were instrumental in evaluating the cultural suitability, acceptability and usability of the original American FEPT version and making recommendations to enhance adaptation of the FEPT-Multiple-Races (FEPTMR)-Shona for use with Shona speaking Zimbabweans (Table 1).

**TABLE 1:** Facial emotion perception test Shona adaptation steps.

Adaptation steps	Adaptation methods	Changes made
Step 1: Stage setting and expert consultation	Setting up an adaptation team Three bilingual graduates, one health worker and one person with lived experience of depression complete the original test. Cognitive interviews conducted at the end of each test.	Created FEPTMR-Shona version 1 with: Simplified English language instructions. Initial translation from English to Shona. Simplified test response input method (i.e. mouse or trackpad).
Step 2: Preliminary content adaptation	Four bilingual primary health workers and two people with lived experience take FEPTMR-Shona version 1. One focus group discussion conducted with six participants and research team. Results discussed with experts in FEPT.	Created FEPTMR-Shona version 2 which: Introduced multi-racial and higher resolution faces of white, black and Asian ancestry replacing all white faces. Changed test fore and background colour to less distracting colours. Replaced primates and ostriches with cows and small birds. Migrated test platform from PsychoPy3 to Pavlovia.
Step 3: Iterative content adaptation with community members	Five participants with lived experience of depression complete FEPTMR-Shona version 2. One focus group discussion with five participants and the research team. Back/forward language translation results validated by FEPT experts.	Created a touchscreen tablet FEPTMR-Shona version 3 with: An interactive pre-test tutorial with instant feedback. Simplified Shona dialect. Shona voice overs of onscreen test instructions. Adjusted speed at which face stimuli and animal controls are presented.
Step 4: Finalising adaptation	Two health workers, two graduates and three participants with lived experience of depression complete FEPTMR-Shona version 3. Cognitive interviews with participants at the end of each test. One health worker, one graduate and one lived experience participant complete the final FEPTMR-Shona version 4. Focus group discussion with three participants and research team.	Finalised FEPTMR-Shona (version 4): Adding onscreen dual language instructions (Shona and English). Repeating test instructions at end of every test block. Accessibility on any modern touchscreen browser and checked for internet coverage compatibility.

*Source:* Adapted from Sit HF, Ling R, Lam AIF, Chen W, Latkin CA, Hall BJ. The cultural adaptation of step-by-step: An intervention to address depression among Chinese young adults. Front Psychiatry. 2020;11:650. https://doi.org/10.3389/fpsyt.2020.00650

FEPTMR, FEPT-multiple-race; FEPT, facial emotion perception test.

### Focus group discussions

The focus groups were facilitated by a bilingual (Shona and English) clinical psychologist with experience in adapting psychological interventions for use in Zimbabwe. These structured discussions provided a platform for participants and the research team to collaboratively evaluate the test’s ecological validity by examining key dimensions such as the clarity of facial stimuli, the appropriateness of emotional labels, the suitability of response options and the ease of understanding test instructions. Participants were also encouraged to identify any facial expressions that appeared ambiguous or culturally incongruent.

To maximise engagement and ensure rich, in-depth discussions, each focus group was deliberately kept small (6–12 participants). This format facilitated dynamic exchanges and deeper exploration of culturally relevant themes. A smaller group size allowed each participant ample opportunity to voice their perspectives, ensuring that critical feedback on cultural and procedural aspects of the FEPT was thoroughly explored.

### Cognitive interviews

Cognitive interviews were conducted by a Zimbabwean clinical psychologist with expertise in designing and implementing computer-based cognitive assessment tools for common mental disorders. These interviews employed a think-aloud approach, where participants verbalised their thought processes immediately after completing each version of the adapted FEPT. This method allowed for a detailed examination of participants’ cognitive and emotional reactions to the test, helping to identify challenges related to emotion recognition, the interpretation of emotional labels and potential biases introduced by the test instructions or test platform.

Additionally, cognitive interviews provided a private setting where participants could disclose difficulties, they might have been reluctant to share in a group context, such as challenges with computer literacy, discomfort with the test’s digital interface or struggles in understanding the test instructions. By allowing participants to express these concerns individually, cognitive interviews ensured that usability barriers were fully captured and addressed in the adaptation process.

### Ethical considerations

This study received ethical approval from the Medical Research Council of Zimbabwe, the Research Council of Zimbabwe on 27 July 2021 (Ref no.: MRCZ/A/2390), and the Joint Research Ethics Committee for the University of Zimbabwe and Parirenyatwa Group of Hospitals. Prior to participation, written informed consent was obtained, ensuring participants were fully aware of the study’s aims and procedures. To maintain participant confidentiality, computer-generated codes were assigned as identifiers, replacing personal information. This safeguarded privacy throughout the research process. All identifiable documents, including signed consent forms, were securely stored in locked filing cabinets within the Department of Mental Health at the University of Zimbabwe. Additionally, participant data were securely managed and stored online using the Pavlovia and Redcap databases. These platforms provided robust security measures to protect sensitive information, ensuring data integrity and confidentiality throughout the study.

## Results

Fifteen participants took part in the adaptation process. The 15 participants comprised four graduates with a background in psychology, counselling and social work, four primary health workers, and seven people with lived experience of depression attending outpatient primary care services. Three FGDs and 12 cognitive interviews were conducted to gather feedback on the suitability and usability of the FEPT throughout the adaptation process. The complementary use of focus groups and cognitive interviews ensured a comprehensive adaptation process, aligning with Bernal’s Ecological Validity Model by integrating both social and individual perspectives on the test’s cultural relevance. Feedback from both methods was systematically incorporated into refinements of the different versions of the FEPT under adaptation.

### Changes to facial emotion perception test language and content (Table 2 and Figure 1)

**FIGURE 1:**
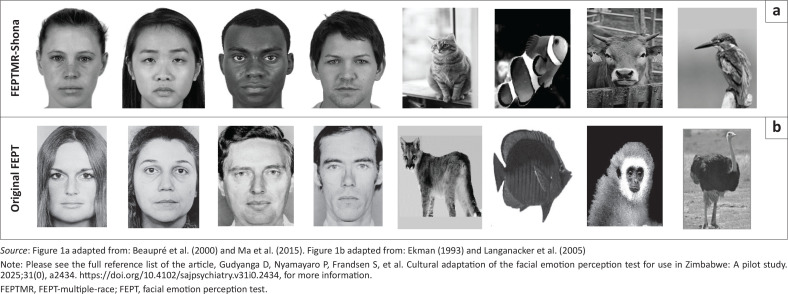
Images of: (a) FEPT-multiple-races-Shona and (b) original facial emotion perception test.^14,31,34,35^

Results from the FGDs emphasised the need for culturally appropriate and universally recognisable stimuli, and for clearer images to aid identification of emotions. This led to the team replacing all 60 white faces, with white, black and Asian ancestry faces of higher resolution from the Chicago Face Database,^34^ and the Montreal Set of Facial Displays of Emotion^35^ (Table 2^14^ and Figure 1^14,31,34,35^). These featured happy, angry, sad, fearful and neutral expressions, with four faces per emotion for each gender and race. Images were cropped to remove cultural indicators like clothing.

**TABLE 2:** Facial emotion perception test original and revised task features.

Feature of FEPT	Original FEPT	FEPTMR-Shona
Language of onscreen test instruction	English	Shona and English
Voiceovers for test instructions	Absent	Present
Time allowed to complete the test	7 min	10 min
Test platform	Computer based test Hosted on PsychoPy3	Any modern touchscreen browser Hosted on Pavlovia
Test response input method/device	Predefined computer keyboard keys	Touchscreen
Face stimuli	60 low-resolution all-white American faces	60 multi-racial high-resolution faces of white, black and Asian ancestry
Face stimuli images	US context (e.g. clothing)	Cropped-out cultural indicators like clothing, present faces only
Animal controls	Low-resolution images of cats, fish, primates, and birds	Higher-resolution images of cats, fish, and birds Replaced primates with cows, ostriches with small birds, and large cats with domesticated cats
Pretest tutorial	Absent	Present

*Source*: Adpated from Langenecker SA, Bieliauskas LA, Rapport LJ, Zubieta J-K, Wilde EA, Berent S. Face emotion perception and executive functioning deficits in depression. J Clin Exp Neuropsychol. 2005;27(3):320–333. https://doi.org/10.1080/13803390490490515720

FEPT, facial emotion perception test; FEPTMR, FEPT-multiple-race; US, United States; min, minutes.

Participant feedback on the lack of universality of original animal images led to replacing primate images with cows, big cats with domesticated cats and ostriches with smaller birds. Additionally, we added instruction sets in multiple modalities (written and voice overs) to enhance comprehension following the recommendation from cognitive interview participants to use simple and clear audio-visual instructions in the local Shona language with hybrid Shona and English responses. We also presented stimuli on less distracting backgrounds. Following these initial adjustments, we translated the FEPT English instructions into Shona, the primary local language, through focus group discussions. We selected a neutral Shona dialect to ensure comprehension across different ethnic groups. We used a hybrid in-person and remote workshop with research assistants, hospital staff and external FEPT experts to compare the back-translated English instructions with the original, leading to further adjustments in Shona.

### Changes to facial emotion perception test methods (Table 2)

The original FEPT version required responses via the computer keyboard. This posed difficulties for participants, who spent more time navigating the keyboard than focusing on the screen. Focus group participants identified several barriers to the usability of the original computer-based FEPT, particularly for primary health care service users receiving depression treatment in Zimbabwe. They noted that most future FEPT users would require at least a moderate level of computer literacy and familiarity with physical keyboards to complete the test successfully. Adaptation participants estimated that approximately 90% of the primary health care patients they encounter, especially those from rural and farming backgrounds, have limited or no exposure to computers, relying primarily on feature phones for digital access. As a result, they expressed concerns that the test’s original format could exclude a significant portion of the intended future FEPT users.

Participants also noted that the rapid presentation of stimuli and limited response time could be challenging for users unfamiliar with computer-based cognitive tasks. They suggested that these time constraints might introduce unintended biases, as difficulties with the task platform, rather than emotion perception ability, could affect test performance. These concerns highlighted the need for modifications to enhance accessibility and usability. Participants also found the original stimuli presentation too fast, further stressing the need to consider future participants’ comfort with technology. Additionally, they emphasised the importance of accounting for cultural value variations in speeded responses when designing the final adapted version of the FEPT. This consideration is particularly crucial for participants in Zimbabwe, as cultural differences can significantly influence the speed at which individuals recognise and respond to facial emotion stimuli. As this may be the first exposure to such tests for many future FEPT users, ensuring cultural sensitivity in the test design will lead to more accurate and equitable assessments of emotional perception across diverse cultural contexts.

In response, we developed a tablet-compatible touchscreen version of the FEPT, which would provide a more familiar phone-like experience. We also introduced a pre-test interactive tutorial to familiarise participants with the task and slow down the stimuli presentation while maintaining the test’s original psychometric properties. Participants reacted positively to the introduction of a touchscreen-compatible version, stating that this would accommodate users with limited to no computer experience. Similarly, extending the response time was widely regarded as beneficial, as it would reduce task-related stress and allow respondents to focus on recognising emotions rather than struggling with the test format.

**FIGURE 2:**
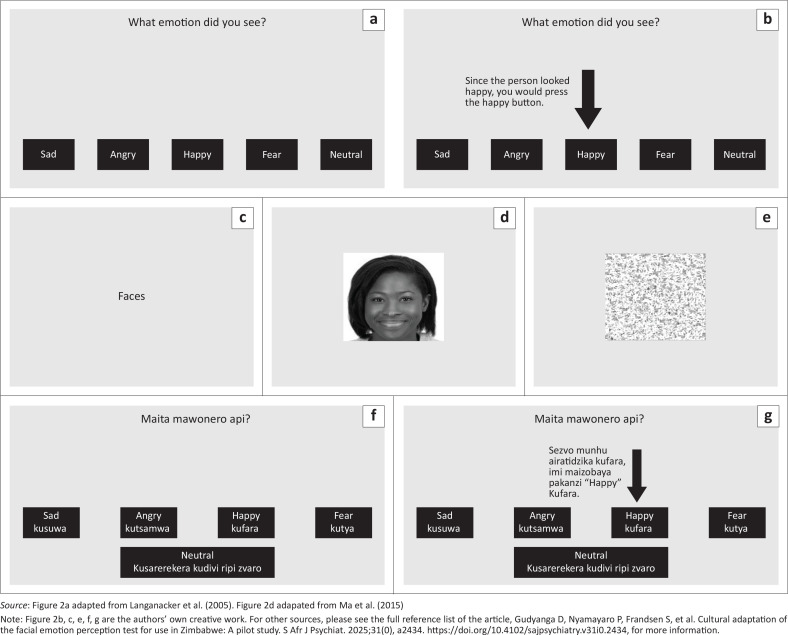
Evolution of facial emotion perception test response screens from original facial emotion perception test to FEPT-multiple-race – Shona: (a) Task response screen for the original FEPT. (b) FEPT with added pre-test interactive tutorial. (c) FEPTMR – Shona category introductory slide indicating whether incoming task is animals or faces. (d) FEPTMR – Shona facial emotion stimuli (e) FEPTMR – Shona mask slide displayed soon after target facial emotion stimuli (f) FEPTMR – Shona onscreen instructions with Shona and English responses (g) FEPTMR – Shona task response screen with pre-test interactive tutorial.

Further, participants reported that the interactive tutorial would also help accommodate varying educational backgrounds by familiarising respondents with the FEPT and boosting their confidence. The tutorial, preceded by an instructional video and accompanied by onscreen instructions, demonstrated the stimuli (both ‘Face’ and ‘Animal’ trials), task setup, expected response and response instructions. This allows participants to practise with real-time task feedback (Figure 2: FEPT response screens). The tutorial can be repeated to enhance comprehension of task rules.

The adapted FEPT Multiracial Shona (FEPTMR-Shona) test includes 60 faces (12 per emotion: happy, sad, fearful, angry, neutral) from white American, black American and Asian ancestry, equally divided by gender. The test consists of 120 alternating ‘Face’ and ‘Animal’ trials, taking 10 minutes to complete. Participants identify emotions or animals from the provided options. Hosted on Pavlovia, it works on most touchscreen devices. Pre-downloaded surveys ensure usability in areas with intermittent internet, and results can be uploaded online or downloaded locally as a comma-separated values file (CSV).

## Discussion

We completed an iterative cultural adaptation of the original version of the FEPT, leading to the first example of a facial emotional perception test that shows promising cultural relevance and usability for Zimbabwean Shona speakers. This is consistent with Garabiles and colleagues’ emphasis on the importance of considering cultural relevance, acceptance, completeness and comprehensibility when adapting tests from one context to another.^36^ As such, the key adaptations of the FEPTMR-Shona included the addition of multi-racial face stimuli, higher resolution photographs of white, black and Asian ancestry faces, extended test time, additional instructions and a user-friendly touchscreen platform. This related to making adjustments in consideration of the socio-economic and limited technological accessibility available in a low-resources context of LMICs, which have the potential to reduce test performance biases related to test functionality.

Burchert and colleagues stress that contextual adaptation of assessment tools should prioritise user experience and usability.^37^ Considering that about half of FEPTMR – Shona users are unfamiliar with computer-delivered cognitive tests and have low computer literacy, especially as about 70% of the Zimbabwean population is rural-based, making up 7–10% of computer users, and about 70% of basic feature phones or simple smartphone users,^38,39^ we extended the test time and added simple, clear instructions to improve comprehensibility and reduce performance biases. We also switched the test platform from typed responses on a keyboard to a touchscreen tablet, addressing the technical literacy barrier highlighted by Burchert and colleagues.^37^ This important consideration was reflected in the distribution of the cultural adaptation participants, who had varying levels of computer literacy. Half of the participants were familiar with computers and/or computer-based tests, ensuring that the adaptation accounted for the differences in computer familiarity levels between rural and urban Zimbabweans.^38,39^

The participant feedback highlighted critical usability concerns regarding the original FEPT format, reinforcing the importance of cultural and contextual adaptation in psychological assessment tools. Consistent with Bernal’s Ecological Validity Model, which emphasises procedural and contextual relevance, participants pointed out that limited computer literacy among primary health care users, particularly those from rural areas, could serve as a major barrier to test completion. Their concerns about task difficulty and response-time constraints align with previous research indicating that digital cognitive assessments may disadvantage individuals with minimal prior exposure to computerised testing environments.

The positive reception of the touchscreen-compatible version and extended response time suggests that these adaptations were both necessary and effective in improving accessibility. By reducing reliance on physical keyboards and easing time constraints, these modifications help ensure that performance on the FEPT reflects emotion perception ability rather than familiarity with digital interfaces. Future research should further evaluate the impact of these changes on test validity and reliability in a larger, more diverse sample.

Our adaptation process carefully considered both technical and language literacy to ensure the acceptability of FEPTMR-Shona among Zimbabwean Shona speakers of varying educational backgrounds. Recognising the fact that Shona speakers are more accustomed to reading English text rather than Shona text, because of the widespread use of English in Zimbabwe, we provided written instructions in both languages and verbal instructions in Shona. This flexibility, as emphasised by Burchert and colleagues,^37^ helps overcome language barriers in digital health tools, which are often only available in English.

We went beyond basic forward and back translations, incorporating dynamic adjustments through bidirectional feedback and prioritising simplicity and clarity in both Shona and English instructions and voiceovers. This approach improves upon traditional cultural adaptations that typically focus only on translation. Additionally, we considered other relevant features for complex studies of facial emotion perception,^40^ like stimuli presentation and comprehensibility of test procedures and instructions. This is in line with recommendations on cultural adaptation considerations around the content and methods of digital health tools such as language translation, simplification and the amount and style of guidance provided by the test.^41^

Same-race and same-sex biases, like enhanced accuracy and more rapid speed of detection, tend to occur in many emotion processing samples and are only partially mitigated by exposure across sexes and cultures. High-resolution multiracial photographs of white, black, and Asian ancestry were incorporated into the FEPTMR-Shona to mitigate this bias, making it easier to see emotions on the faces of people from different racial heritage. A key consideration, as Elfenbein and colleagues^22^ demonstrated, is that the more a person is familiar with the culture, the more likely their facial expressions are correctly recognised. Thus, the adaptation process carefully considered the influence of cultural familiarity on emotion perception.

## Limitations

Grounded in the Ecological Validity Model, this study provides preliminary work on the cultural adaptation of the Facial Emotion Perception Test for Shona-speaking Zimbabweans. Although the small sample size and purposive sampling approach ensured in-depth input from key stakeholders, including mental health professional graduates, primary health workers and individuals with lived experience of depression, the sample was not fully representative of the broader Zimbabwean population. This limitation affects the extent to which the findings can be generalised to the wider population or different cultural contexts. Future studies should involve larger, more diverse samples to enhance the generalisability and psychometric validation of the adapted Facial Emotion Perception Test. This initial version of the FEPTMR–Shona now needs to undergo psychometric testing in Zimbabwe. Some of the adaptations, especially the language of verbal instruction, are appropriate only for the local Zimbabwean Shona context.

## Conclusion

The Facial Emotion Perception Test (FEPTMR–Shona) for Zimbabwean Shona speakers appears promising for use with Zimbabwean Shona speakers. The process marks a novel advancement in developing culturally appropriate emotion processing measures. The changes made to test language, content and methods enhance its applicability in a low-resource African setting. Future studies should focus on conducting large-scale psychometric testing of the reliability and validity of the FEPTMR–Shona across a representative sample of people with and without depression in Zimbabwe. Reliability would include, for instance, inter-rater and test–retest reliability,^42,43^ and validity would focus on construct validity.^44^ Once shown to have acceptable psychometric properties, we anticipate embedding the FEPT into clinical trials of depression treatment to see if it can predict the response to psychological and/or antidepressant treatment better than existing symptom-based, demographic and socioeconomic variables. If its utility is confirmed, it could later be recommended for clinical use in programmes such as the Friendship Bench.^45^
